# Supplementary material for: Semi-Natural Superabsorbents Based on Starch-g-poly(acrylic acid): Modification, Synthesis and Application
Source: Polymers (Basel). 2020 Aug 10;12(8):1794. doi: 10.3390/polym12081794 (PMC7464871; doi:10.3390/polym12081794)
Supplement: Supplementary file 1 [file polymers-12-01794-s001.pdf]

Supplementary materials

Semi-natural superabsorbent based on starch-g-poly(acrylic acid). Modification, synthesis and application.

Elżbieta Czarnecka, Jacek Nowaczyk

1. Thermogravimetry.

Analyzes were carried out using a Simultaneous TGA-DTA thermal-analyzer type SDT 2960 Simultaneous TGA-DTA from TA Instruments, Champaign, IL, USA) at temperatures from 20°C to 1000°C, at a speed of 10°C·min^{-1}, under air atmosphere with samples of ca. 5-12 mg. Thermogravimetric curves were obtained which depend on the percentage changes in mass of the analyzed sample as a function of increasing temperature. For all components of the diapers under consideration, the beginning and end temperatures of each transformation associated with the change in weight were determined. The total mass loss of the sample is equal to the peak area on the curve. Recorded thermograms were analyzed using TA Universal Analysis Software.

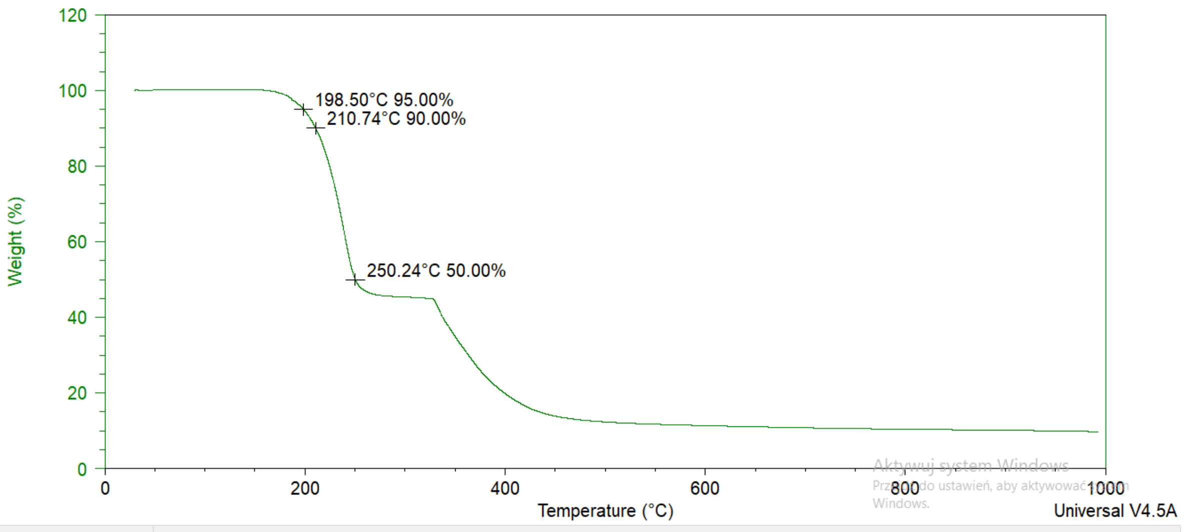

Figure S1. TGA of N, N′ - methylenebisacrylamide (MBA).

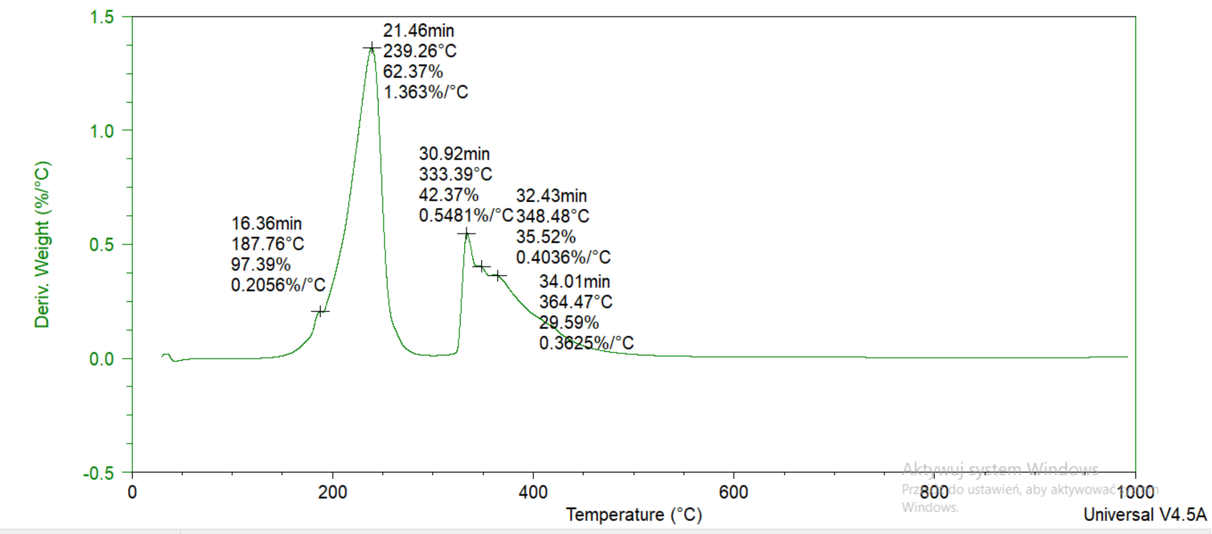

Figure S2. DTA of N, N' - methylenebisacrylamide (MBA).

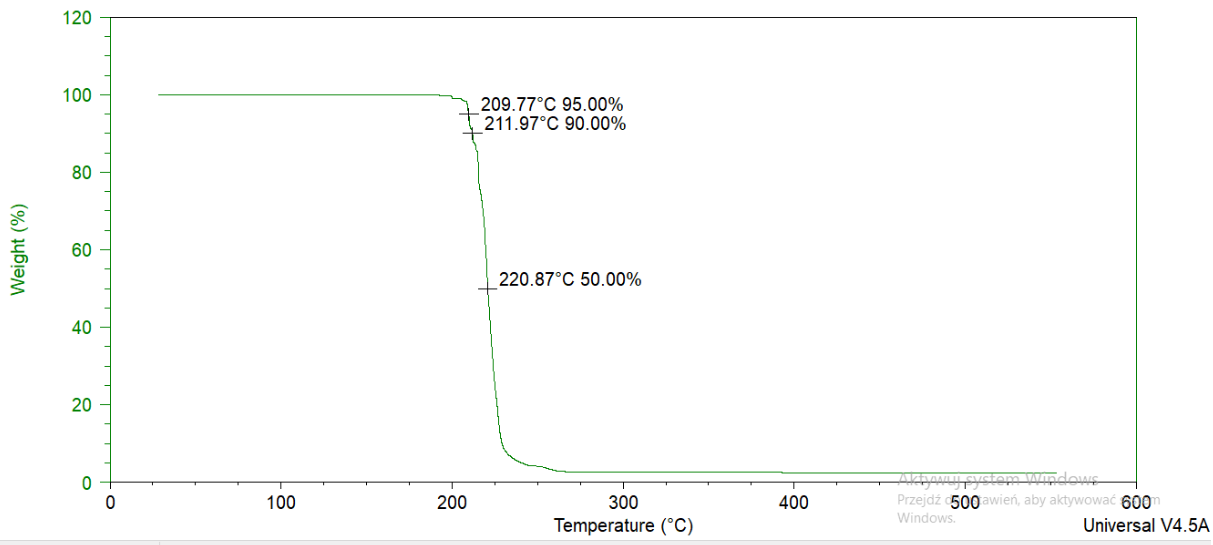

Figure S3. TGA of ceric ammonium nitrate (CAN).

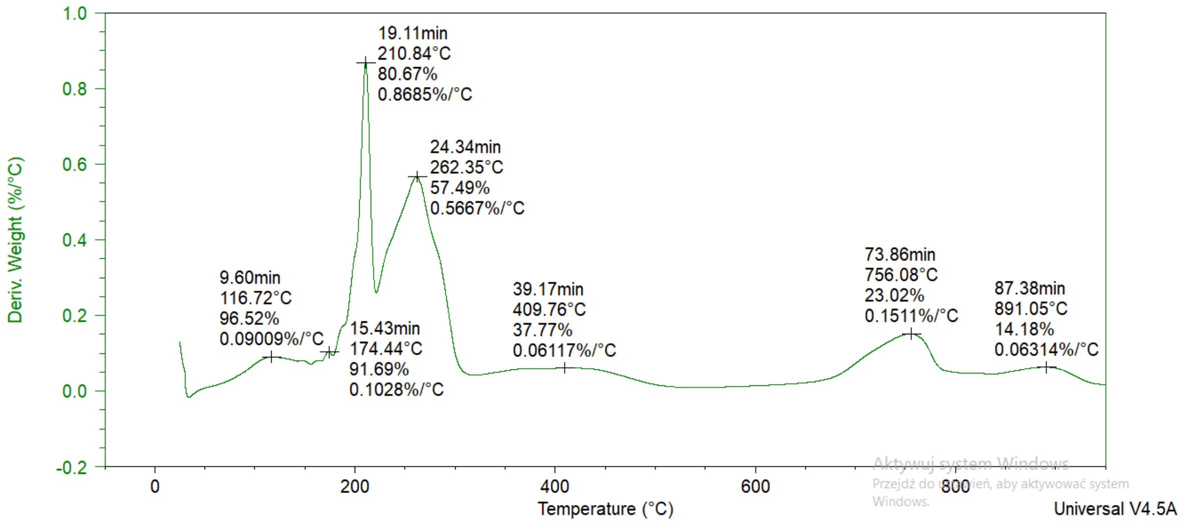

Figure S4.DTA of ceric ammonium nitrate (CAN).

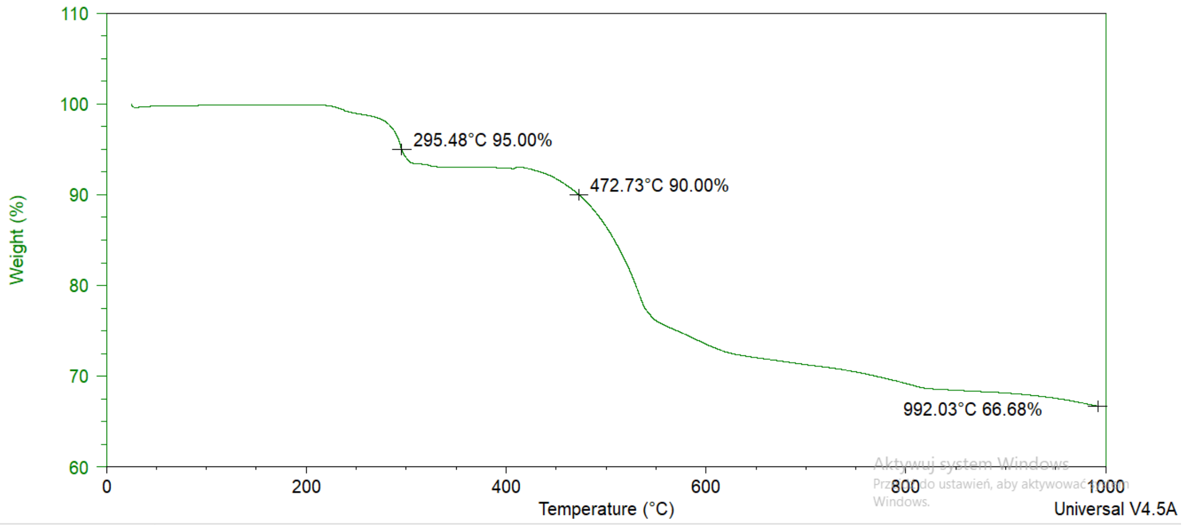

Figure S5. TGA of potassium persulfate (KPS).

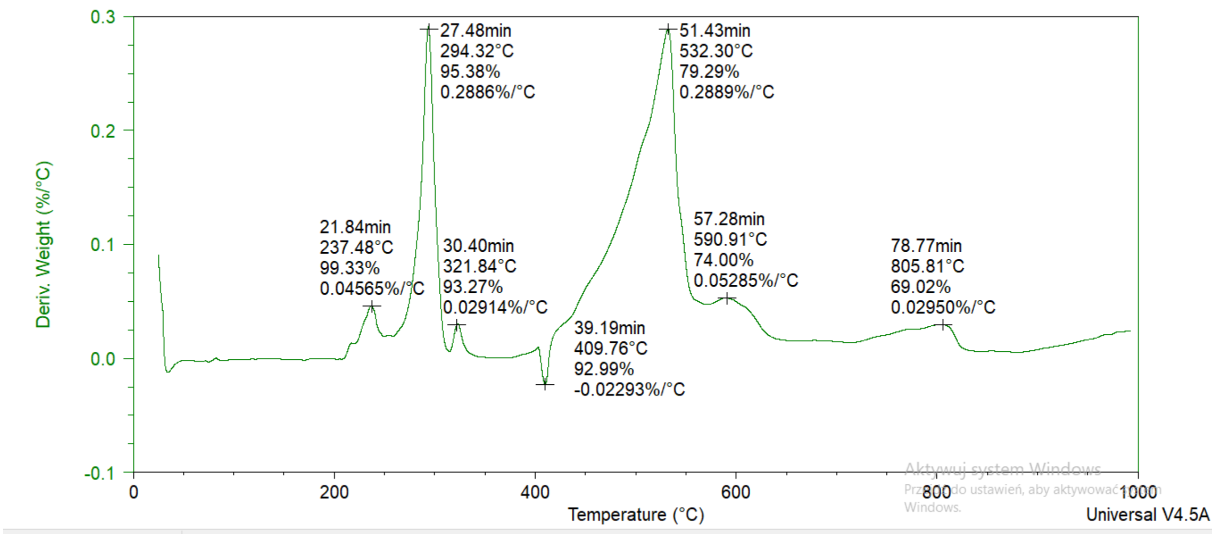

Figure S6. DTA of potassium persulfate (KPS).

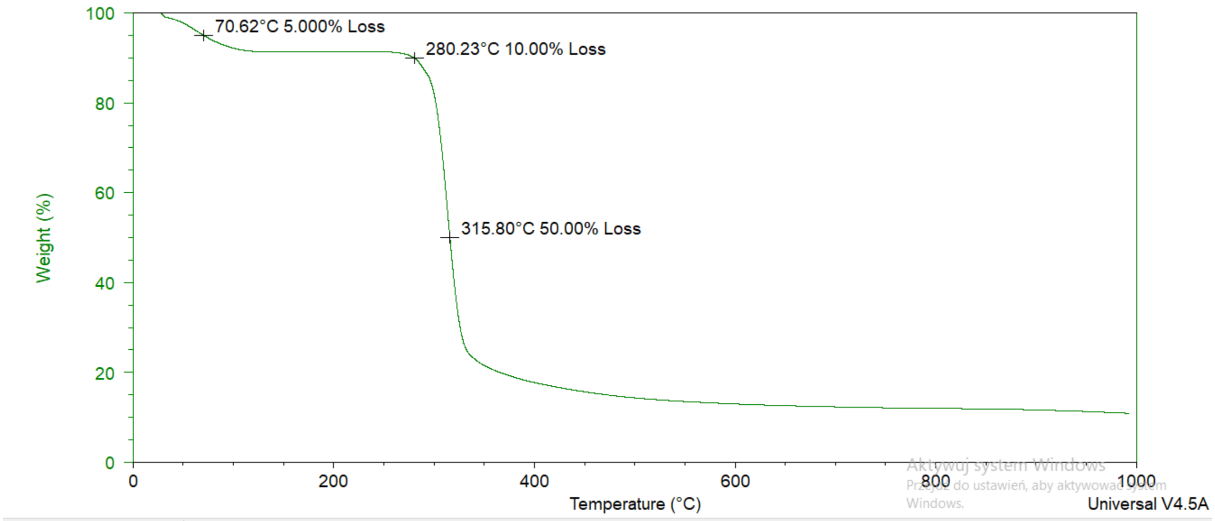

Figure S7. TGA of corn starch (CS).

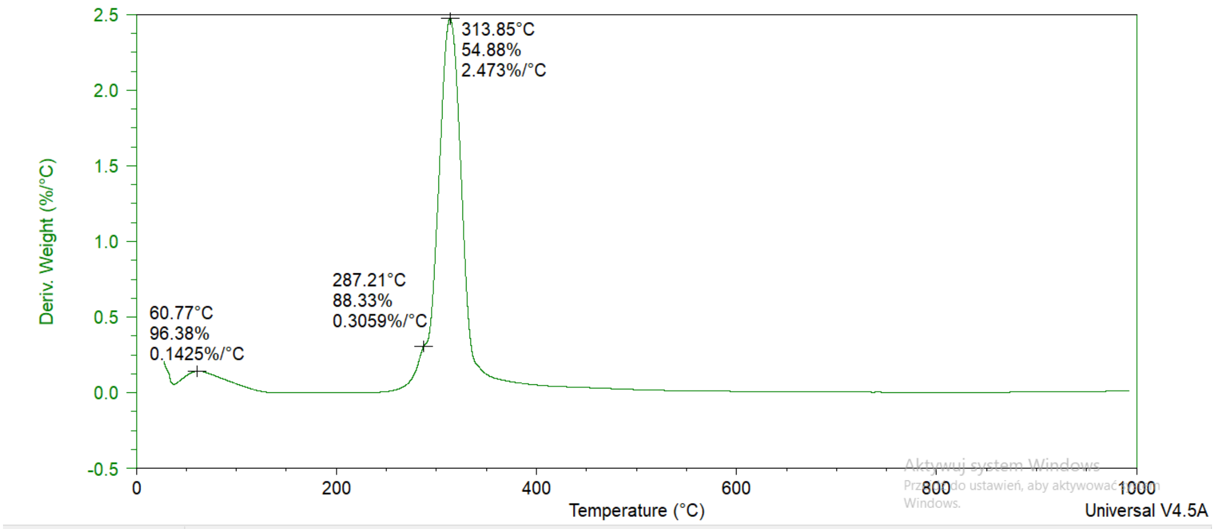

Figure S8. DTA of corn starch (CS).

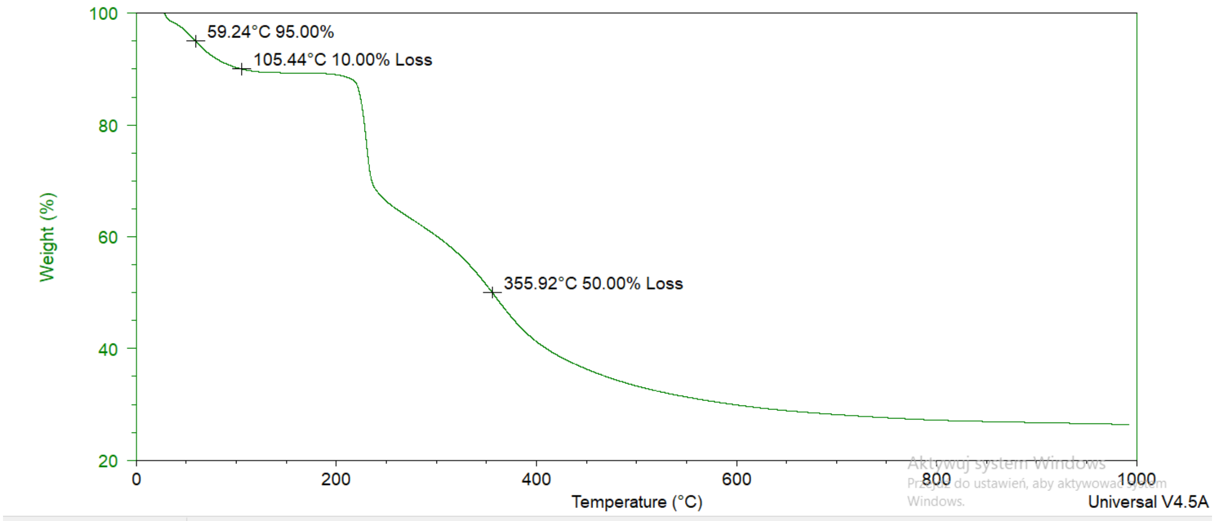

Figure S9. TGA of soluble starch (SS).

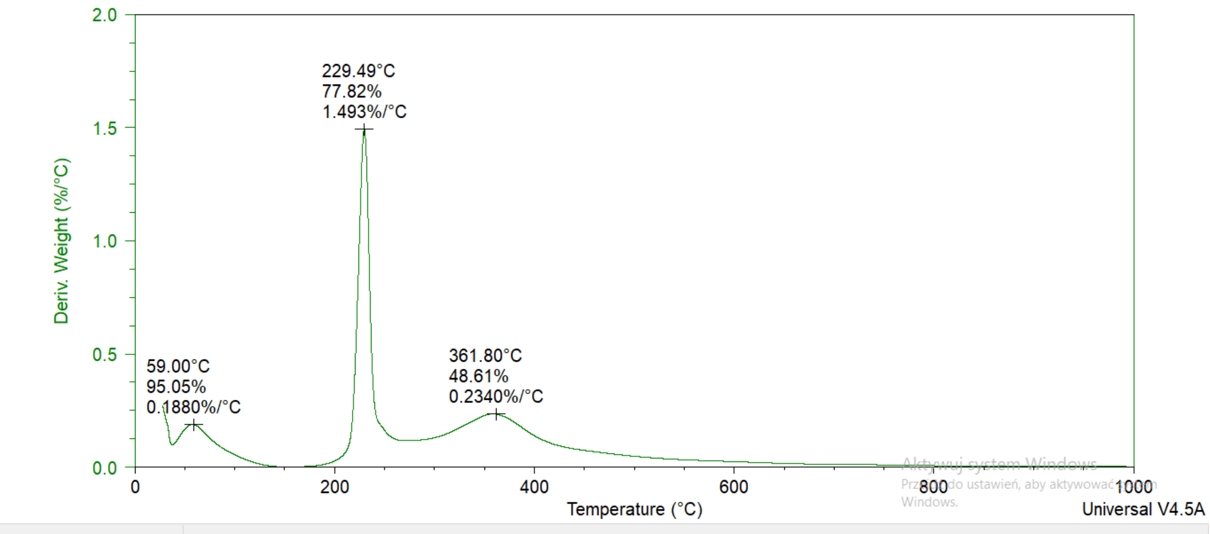

Figure S10. DTA of soluble starch (SS).

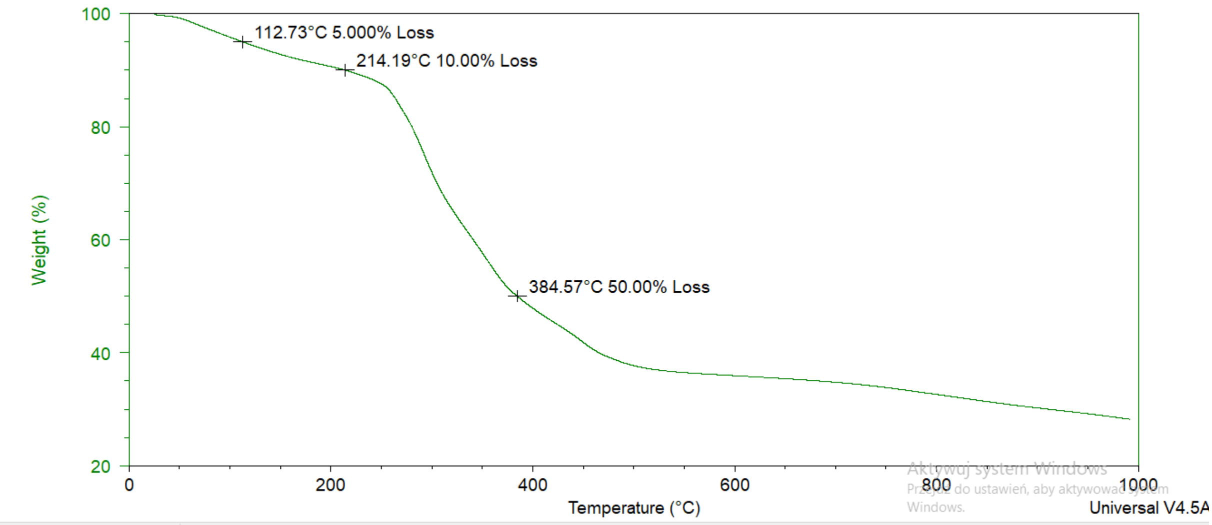

Figure S11. TGA of soluble starch-g-poly(acrylic acid) with potassium persulfate (SS-g-PAA (KPS)).

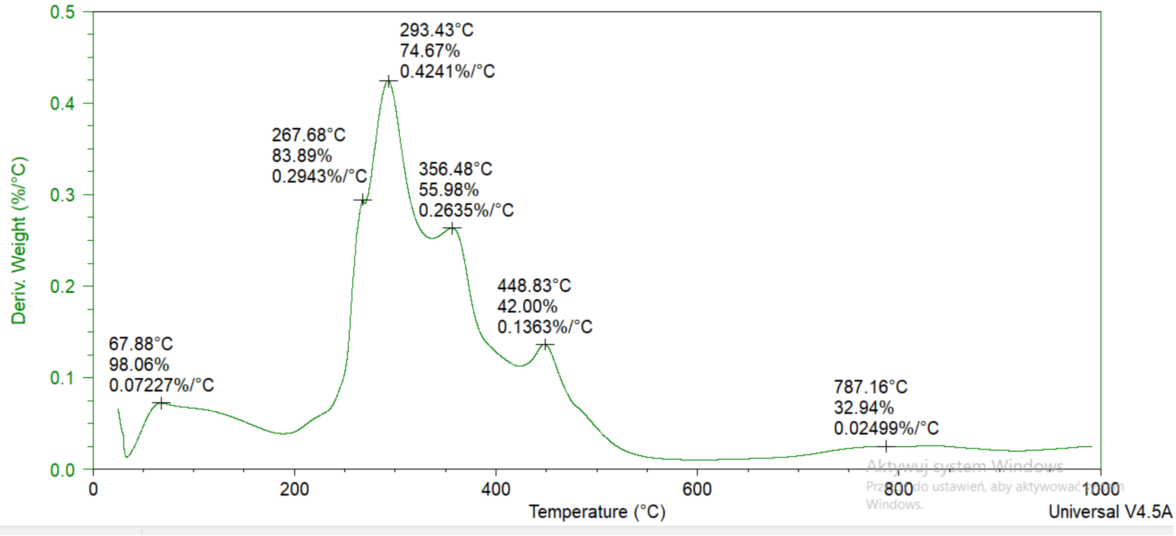

Figure S12. DTA of soluble starch-g-poly(acrylic acid) with potassium persulfate (SS-g-PAA (KPS)).

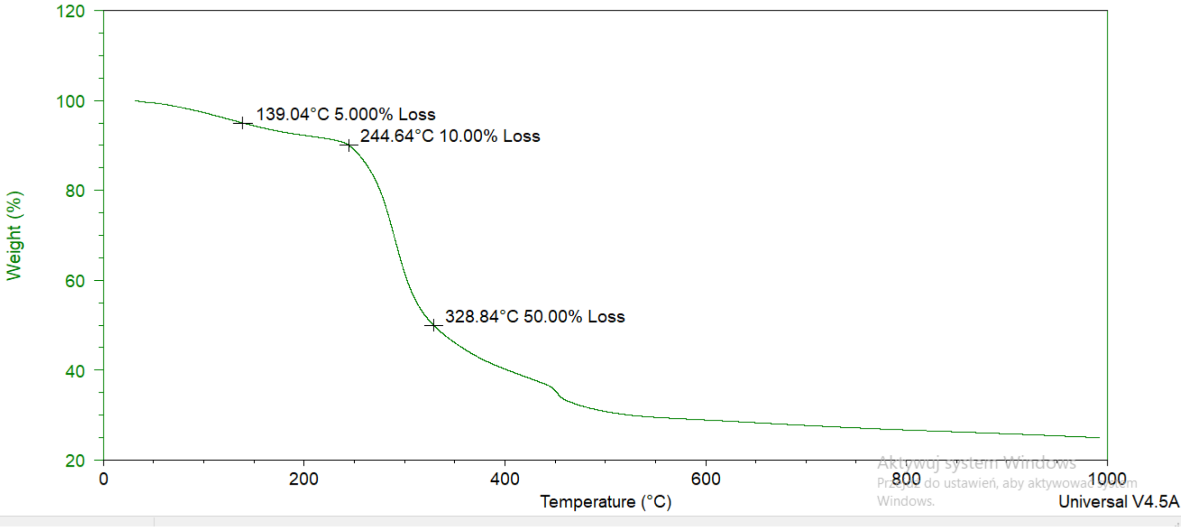

Figure S13. TGA of soluble starch-g-poly(acrylic acid) with ceric ammonium nitrate ((SS-g-PAA(12/CAN)).

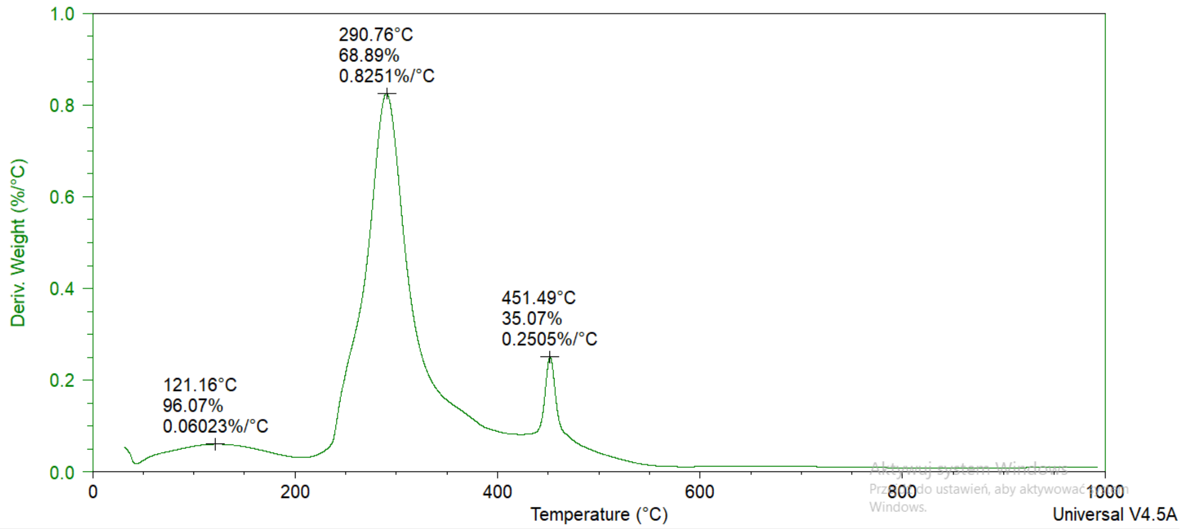

Figure S14. DTA of soluble starch-g-poly(acrylic acid) with ceric ammonium nitrate ((SS-g-PAA(12/CAN)).

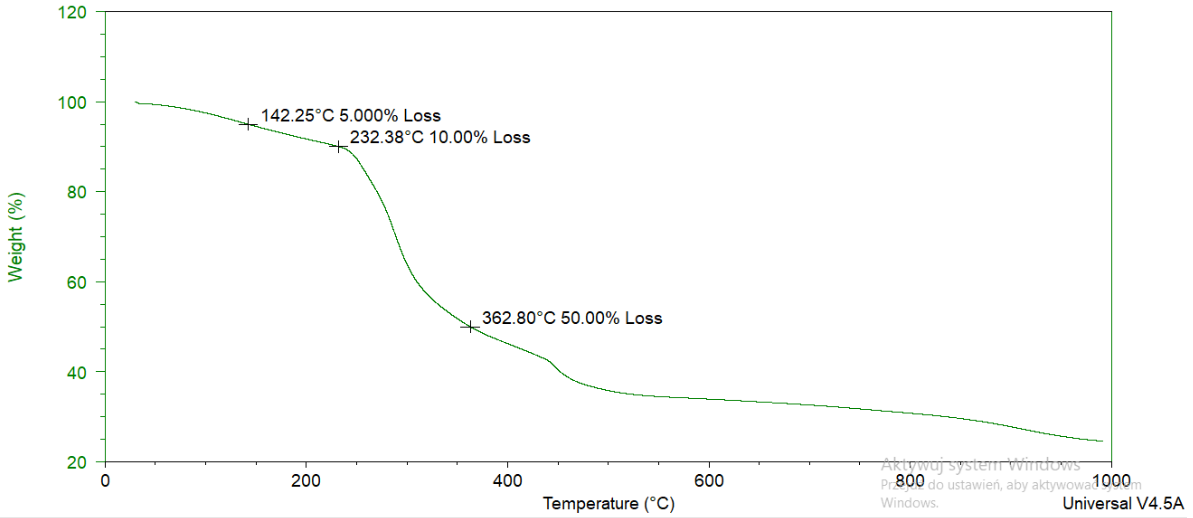

Figure S15. TGA of soluble starch-g-poly(acrylic acid) with ceric ammonium nitrate ((SS(12)-g-PAA(CAN)).

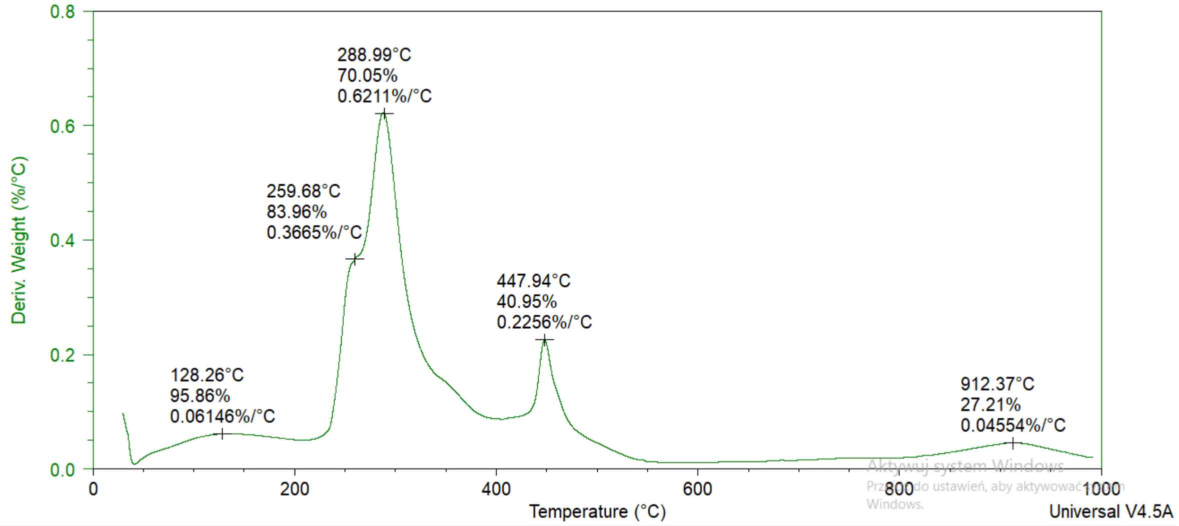

Figure S16. DTA of soluble starch-g-poly(acrylic acid) with ceric ammonium nitrate ((SS(12)-g-PAA(CAN)).

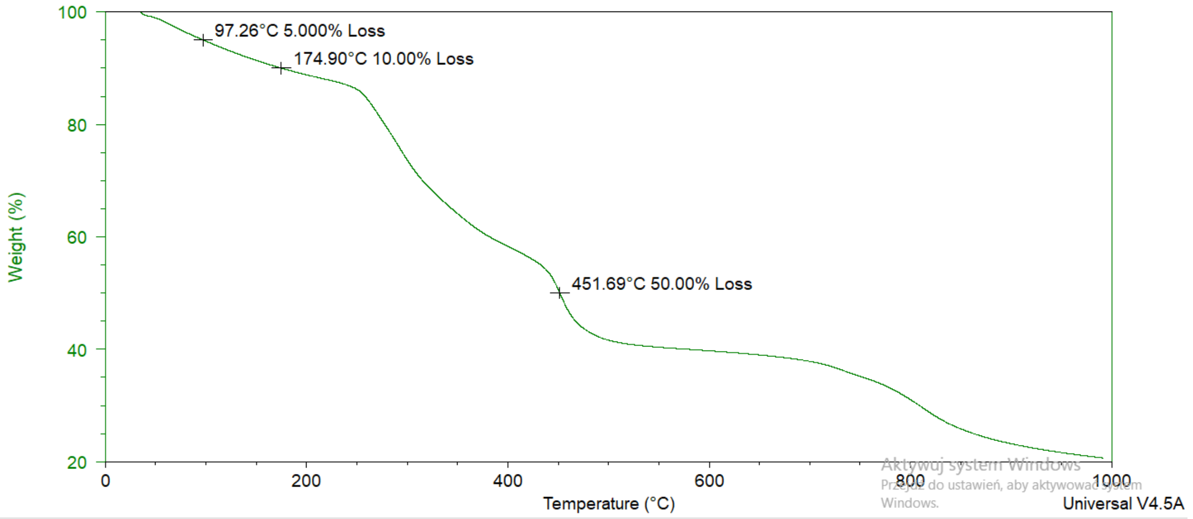

Figure S17. TGA of soluble starch-g-poly(acrylic acid) with ceric ammonium nitrate (SS-g-PAA (CAN)).

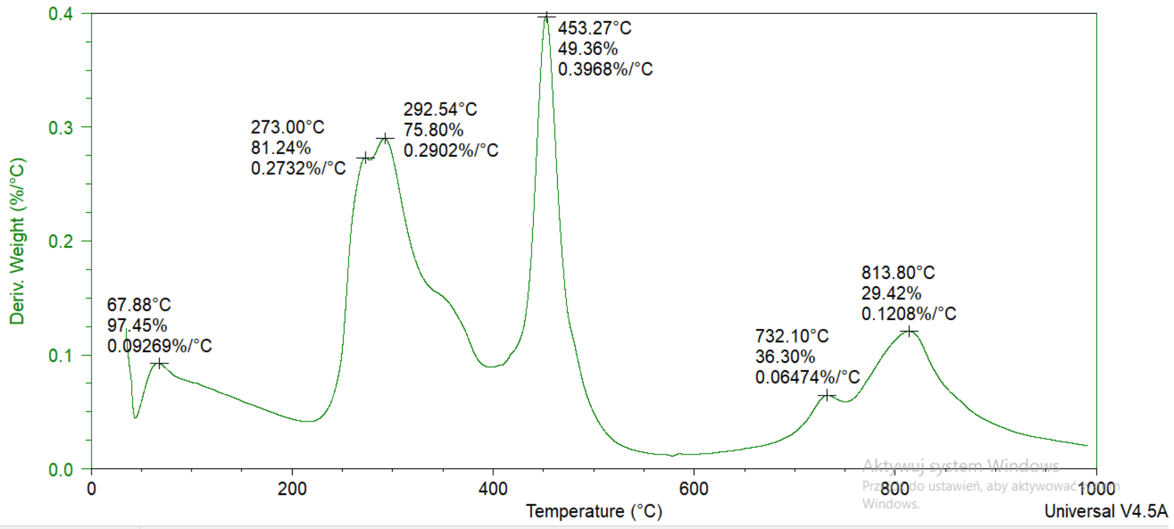

Figure S18. DTA of soluble starch-g-poly(acrylic acid) with ceric ammonium nitrate (SS-g-PAA (CAN)).

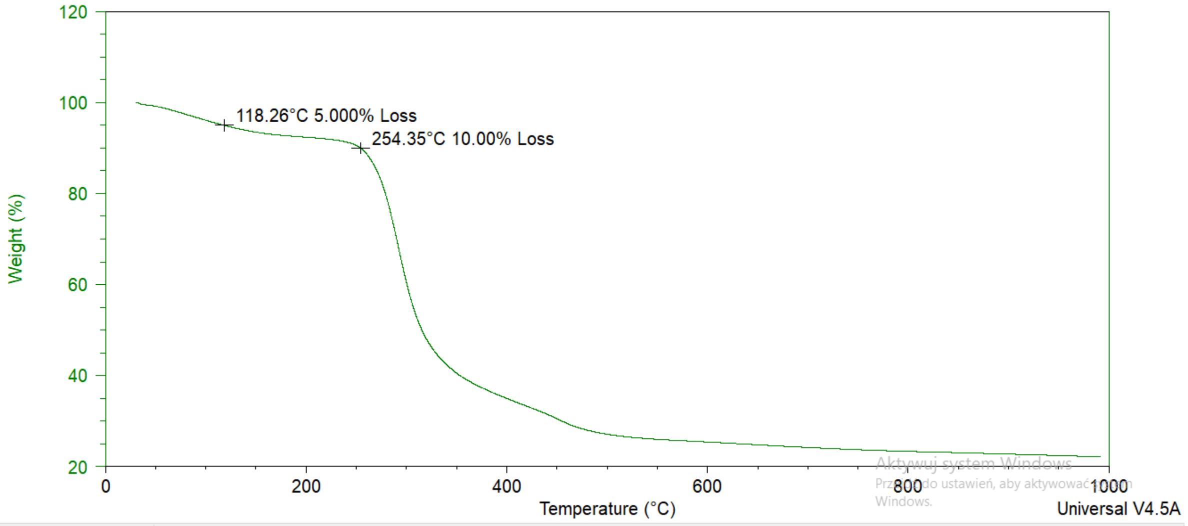

Figure S19. TGA of corn starch-g-poly(acrylic acid) with ceric ammonium nitrate, urea and NaOH.

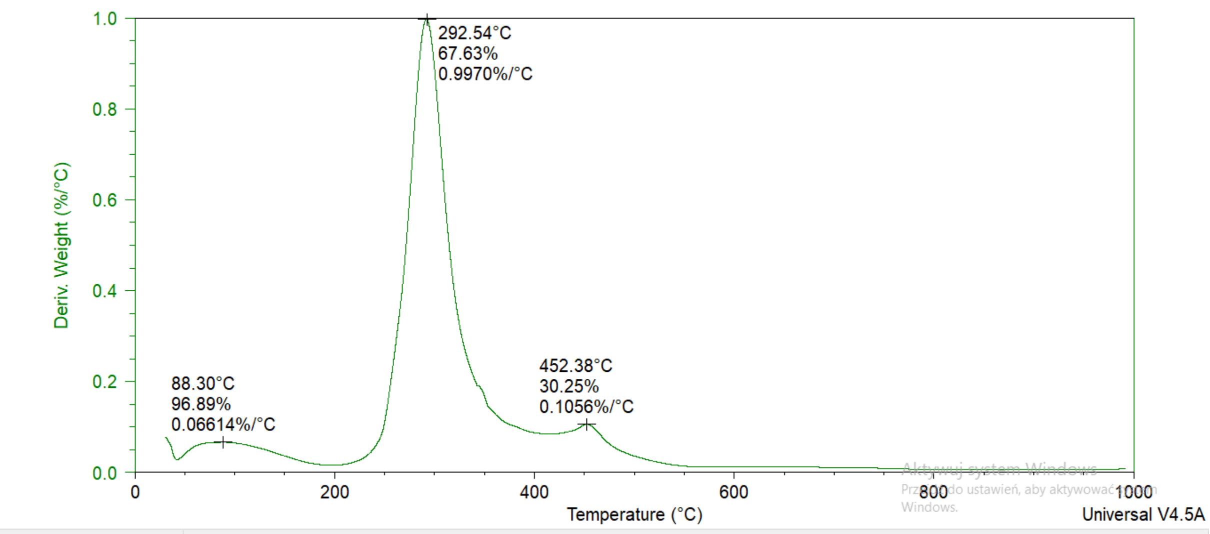

Figure S20. DTA of corn starch-g-poly(acrylic acid) with ceric ammonium nitrate, urea and NaOH.

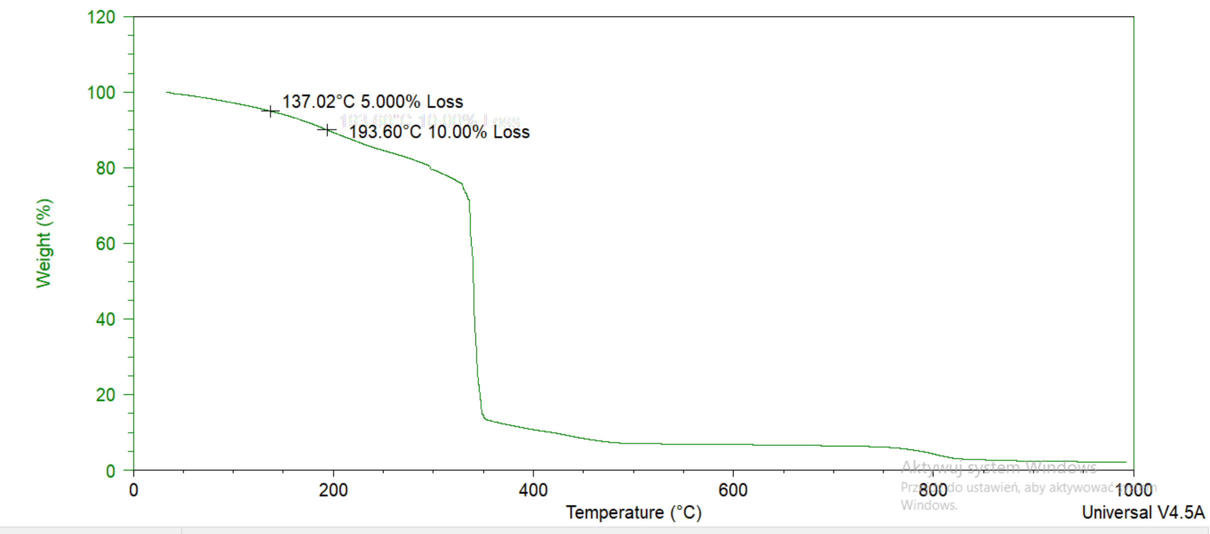

Figure S21. TGA of corn starch-g-poly(acrylic acid) with potassium persulfate, urea and NaOH.

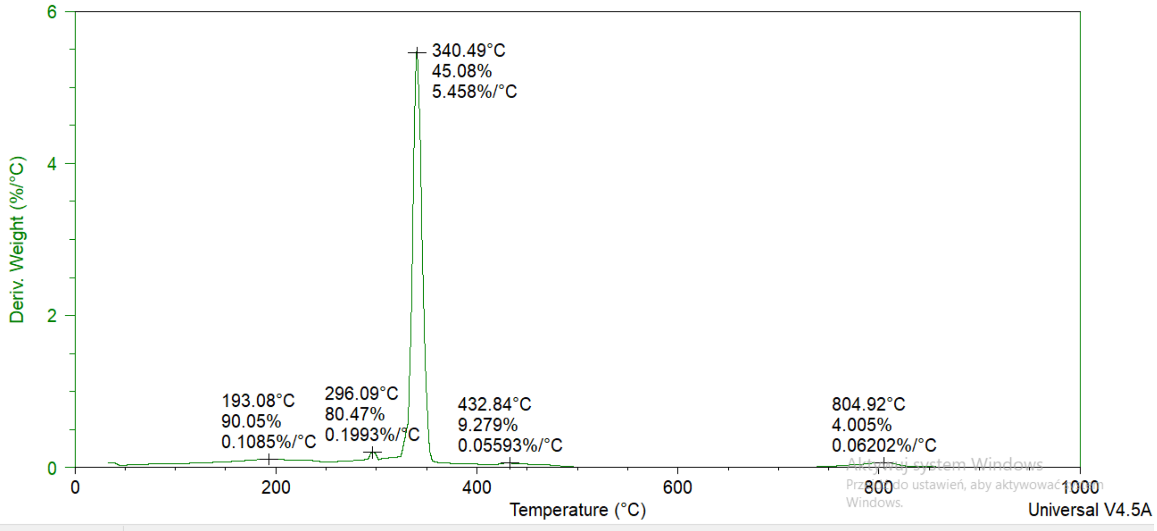

Figure S22. DTA of corn starch-g-poly(acrylic acid) with potassium persulfate, urea and NaOH.

2. Fourier Transform Infrared Spectroscopy

Fourier transmission infrared spectroscopy (FTIR) was used to characterize the presence of specific chemical groups in the materials. FTIR spectra were obtained using a Bruker Vertex 70V spectrometer (Bruker Optoc GmbH, Ettlingen, Germany) in the wave number range from 4000 cm^{-1} to 400 cm^{-1}, for 16 scans with a resolution of 4 cm^{-1}. FTIR spectra have been normalized and the main vibration bands have been associated with chemical groups. The basic components of the product, i.e. excited chemical bonds, absorbed infrared light with a frequency typical for given chemical bonds. All spectra were analyzed using OPUS 7.5 software (Bruker Optoc GmbH, Ettlingen, Germany).

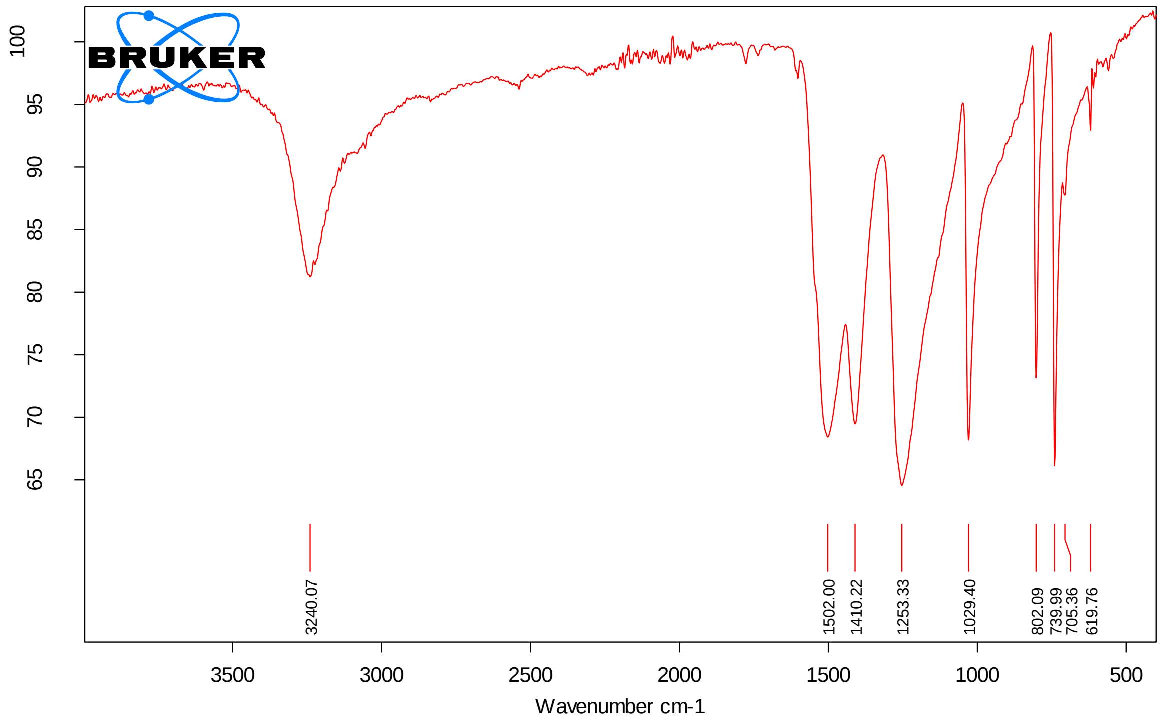

C:\Users\Lenovo\Desktop\Obliczenia\IR syntezy SAP\CAN.0	CAN	Instrument type and / or accessory	07.02.2020

Page 1/1

Figure S23. FTIR spectra of ceric ammonium nitrate (CAN).

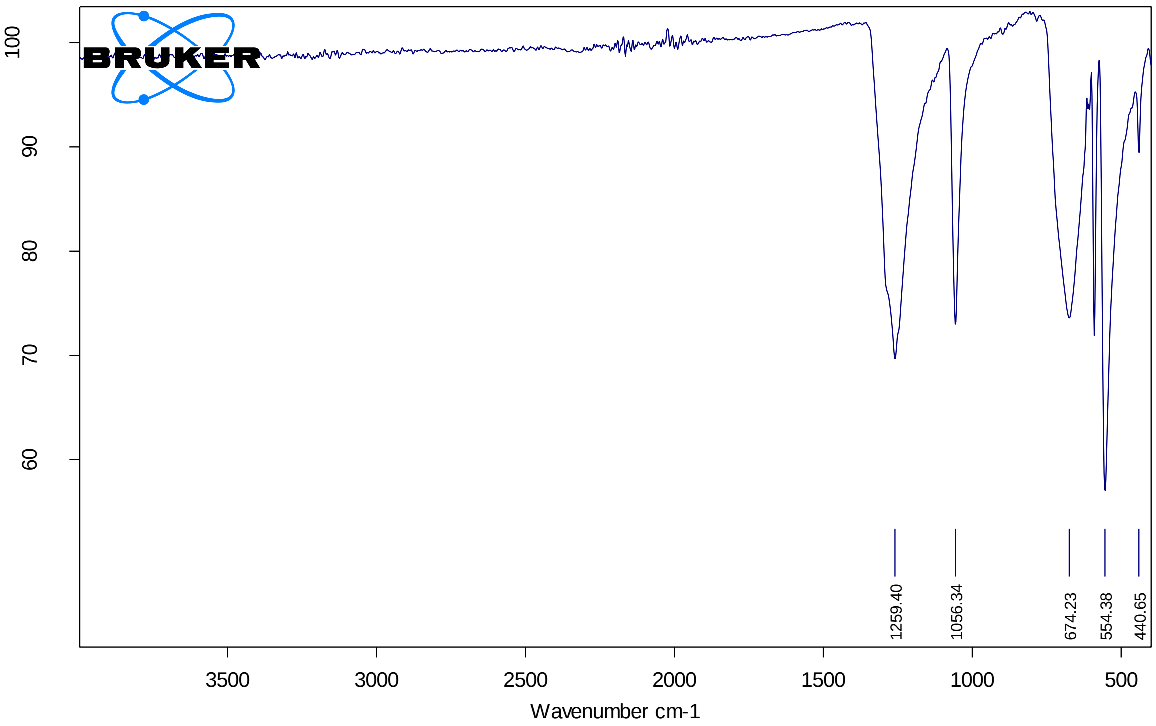

C:\Users\Lenovo\Desktop\Obliczenia\IR syntezy SAP\KPS.0	KPS	Instrument type and / or accessory	07.02.2020

Page 1/1

Figure S24. FTIR spectra of potassium persulfate (KPS).

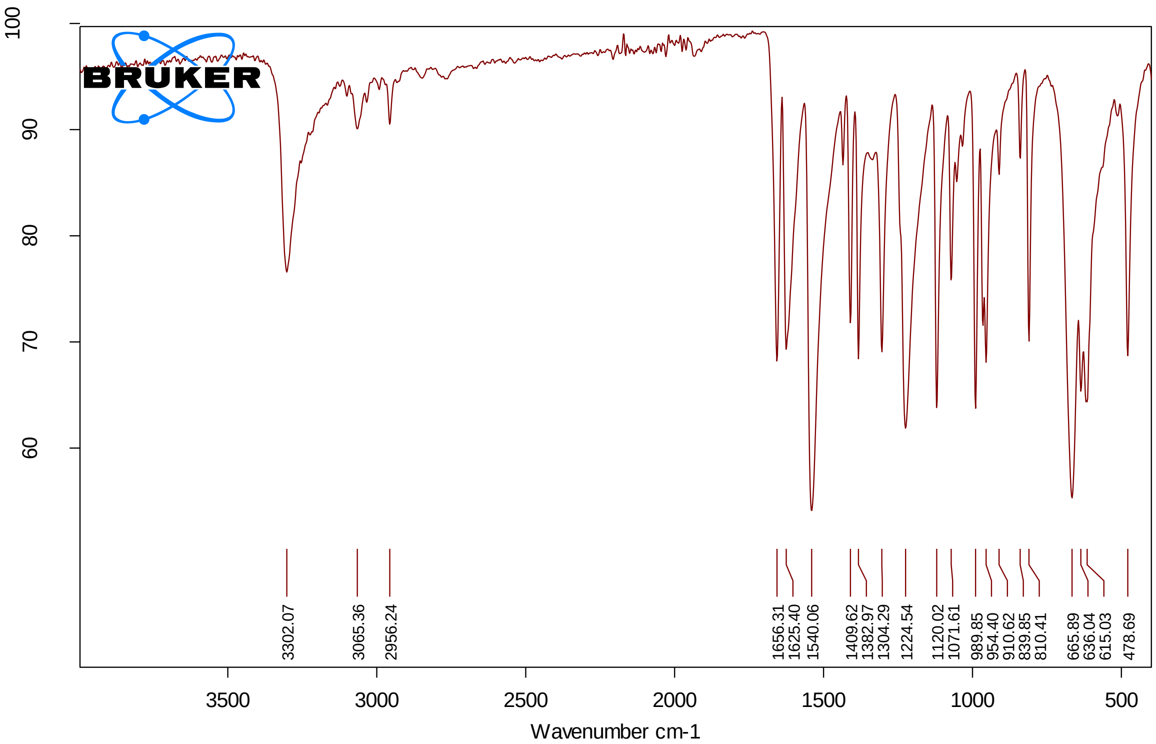

C:\Users\Lenovo\Desktop\Obliczenia\IR syntezy SAP\MBA.0	MBA	Instrument type and / or accessory	07.02.2020

Page 1/1

Figure S25. FTIR spectra of N, N′- methylenebisacrylamide (MBA).

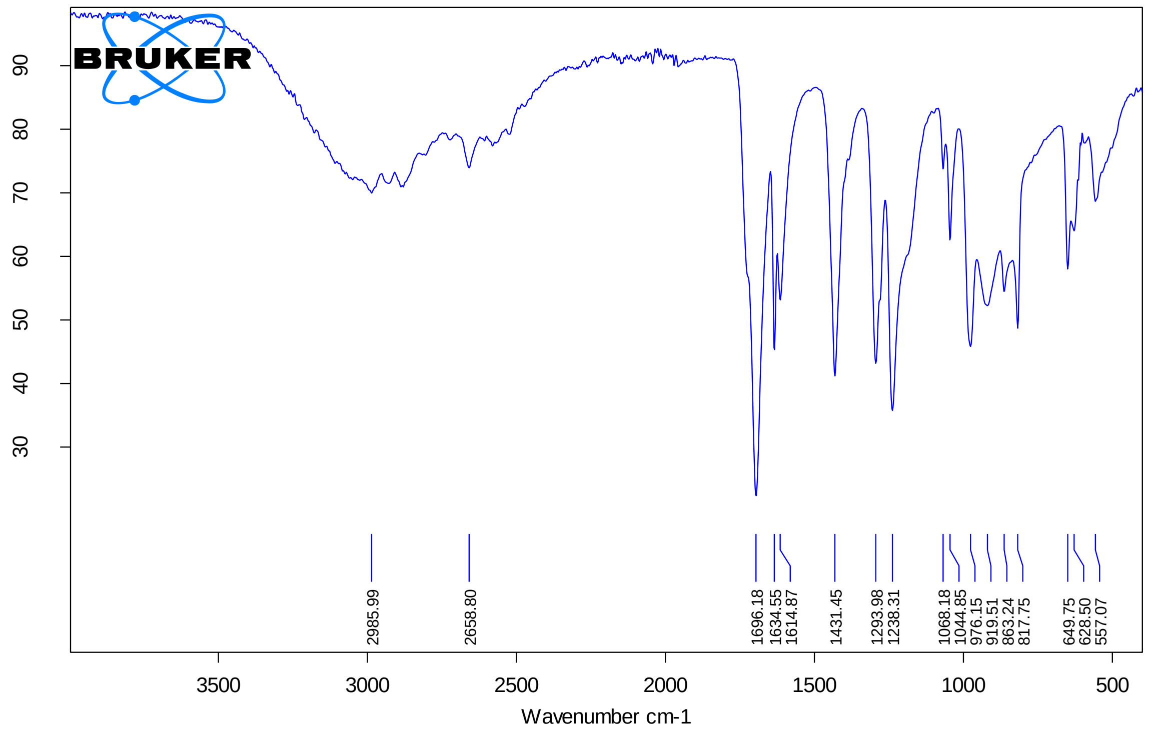

C:\Users\Lenovo\Desktop\Obliczenia\IR syntezy SAP\AA.0 AA Instrument type and / or accessory	07.02.2020

Page 1/1

Figure S26. FTIR spectra of acrylic acid (AA).

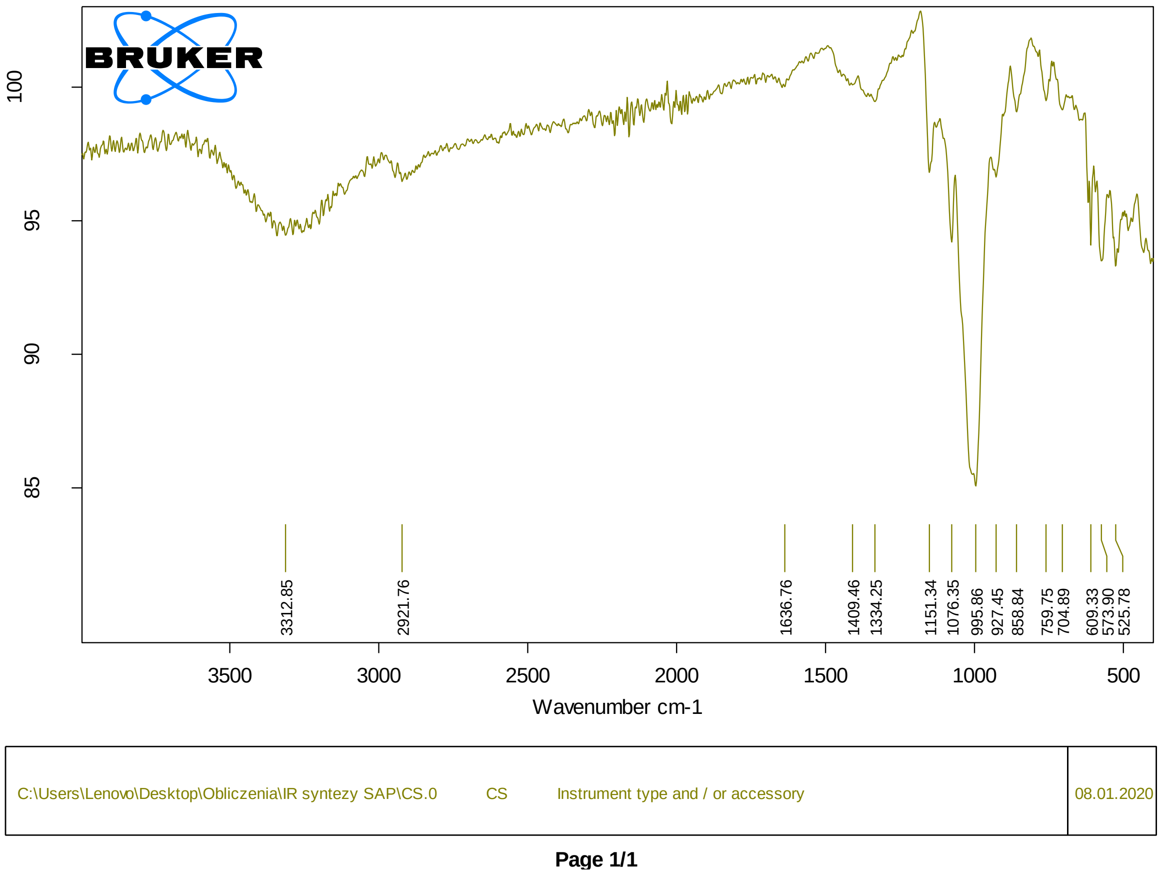

Figure S27. FTIR spectra of corn starch (CS).

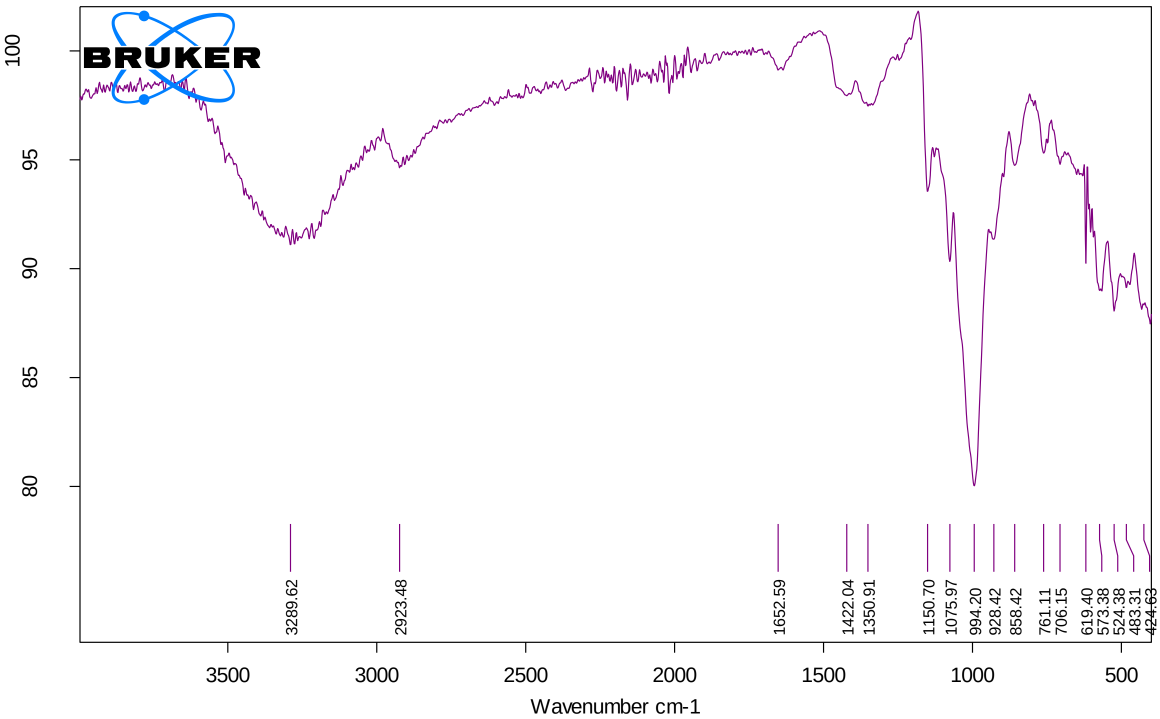

C:\Users\Lenovo\Desktop\Obliczenia\IR syntezy SAP\SS.0	SS	Instrument type and / or accessory	08.01.2020

Page 1/1

Figure S28. FTIR spectra of soluble starch (SS).

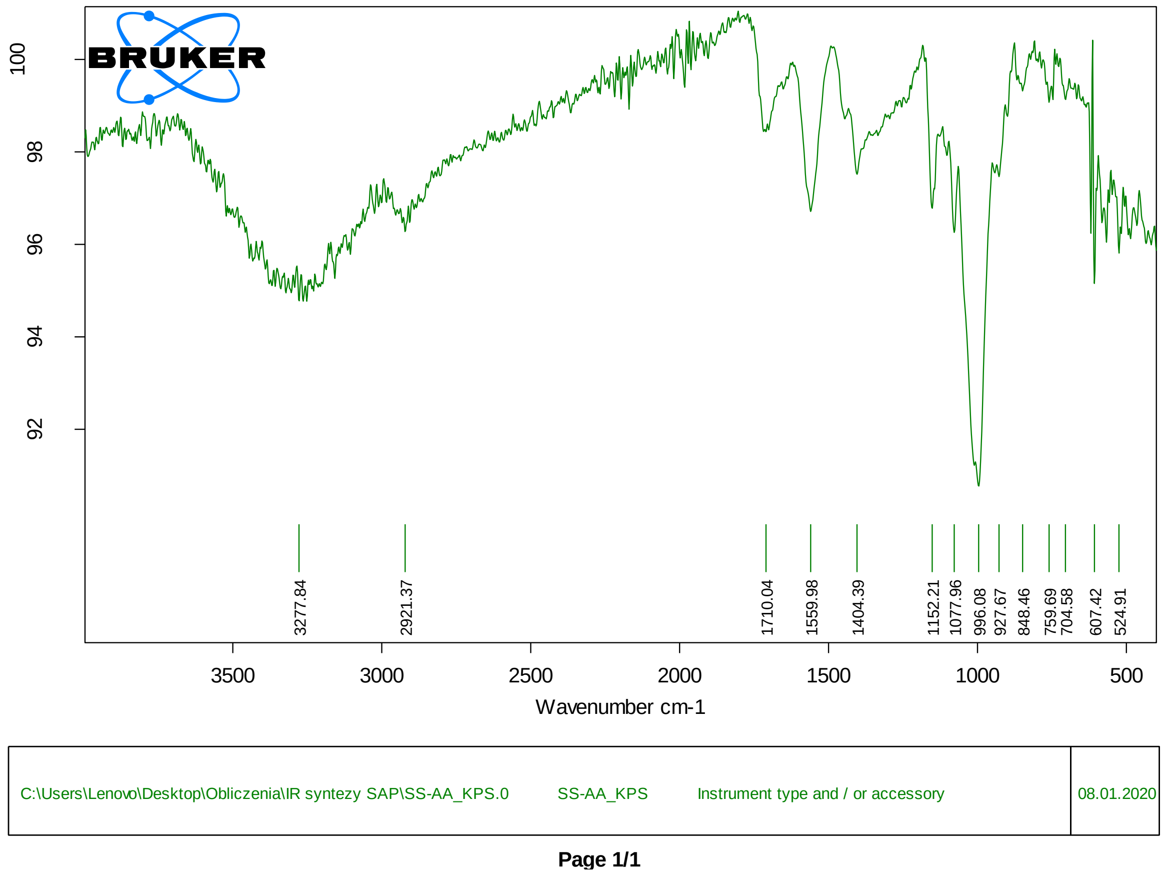

Figure S29. FTIR spectra of soluble starch-g-poly(acrylic acid) with potassium persulfate (SS-g-PAA/KPS).

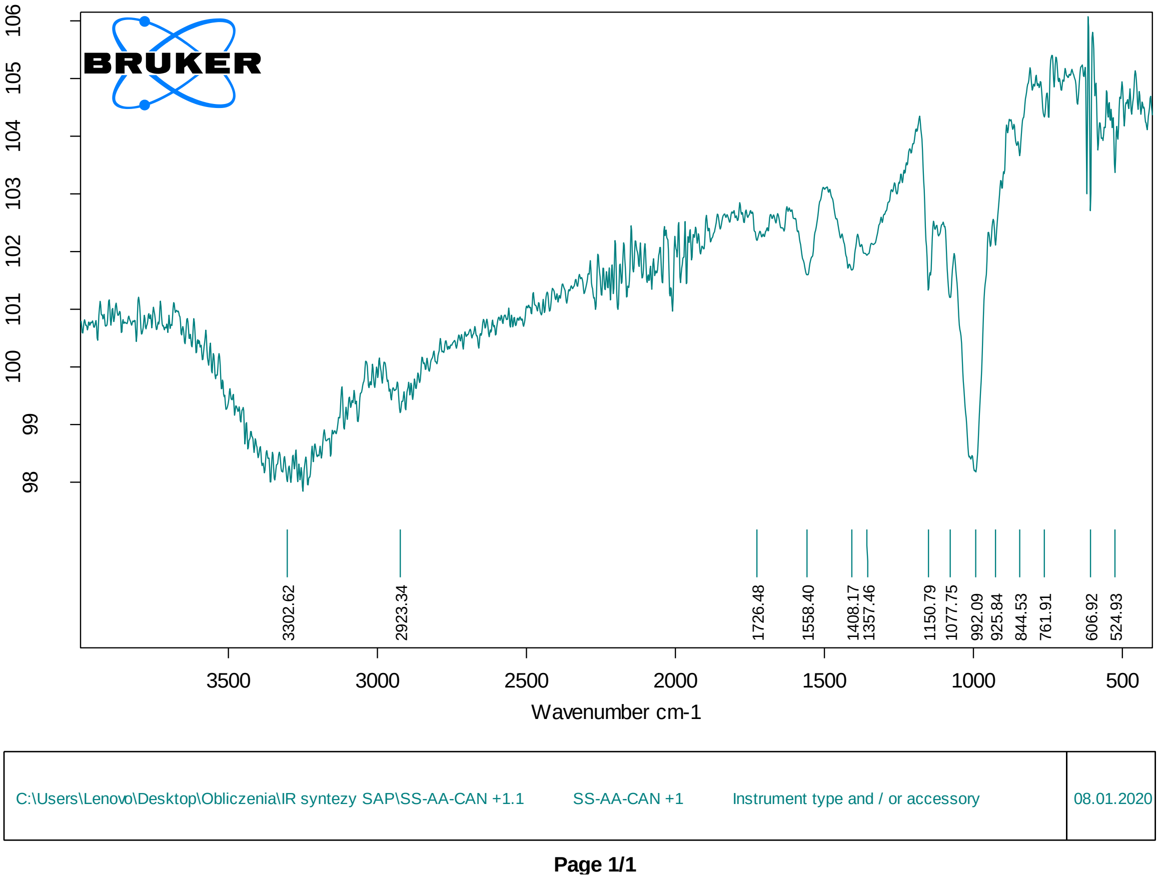

Figure S30. FTIR spectra of soluble starch-g-poly(acrylic acid) with ceric ammonium nitrate ((SS-g-PAA(12/CAN)).

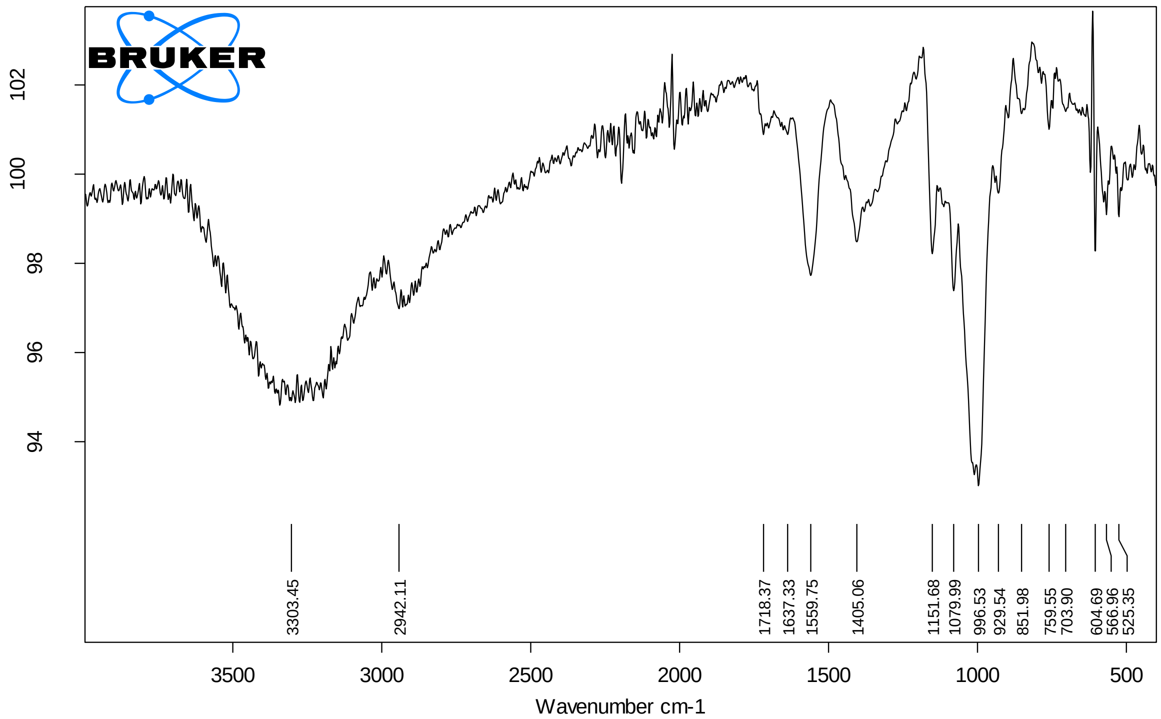

C:\Users\Lenovo\Desktop\Obliczenia\IR syntezy SAP\SS-AA-CAN 1.0 SS-AA-CAN 1 Instrument type and / or accessory	08.01.2020

Page 1/1

Figure S31. FTIR spectra of soluble starch-g-poly(acrylic acid) with ceric ammonium nitrate ((SS(12)-g-PAA (CAN)).

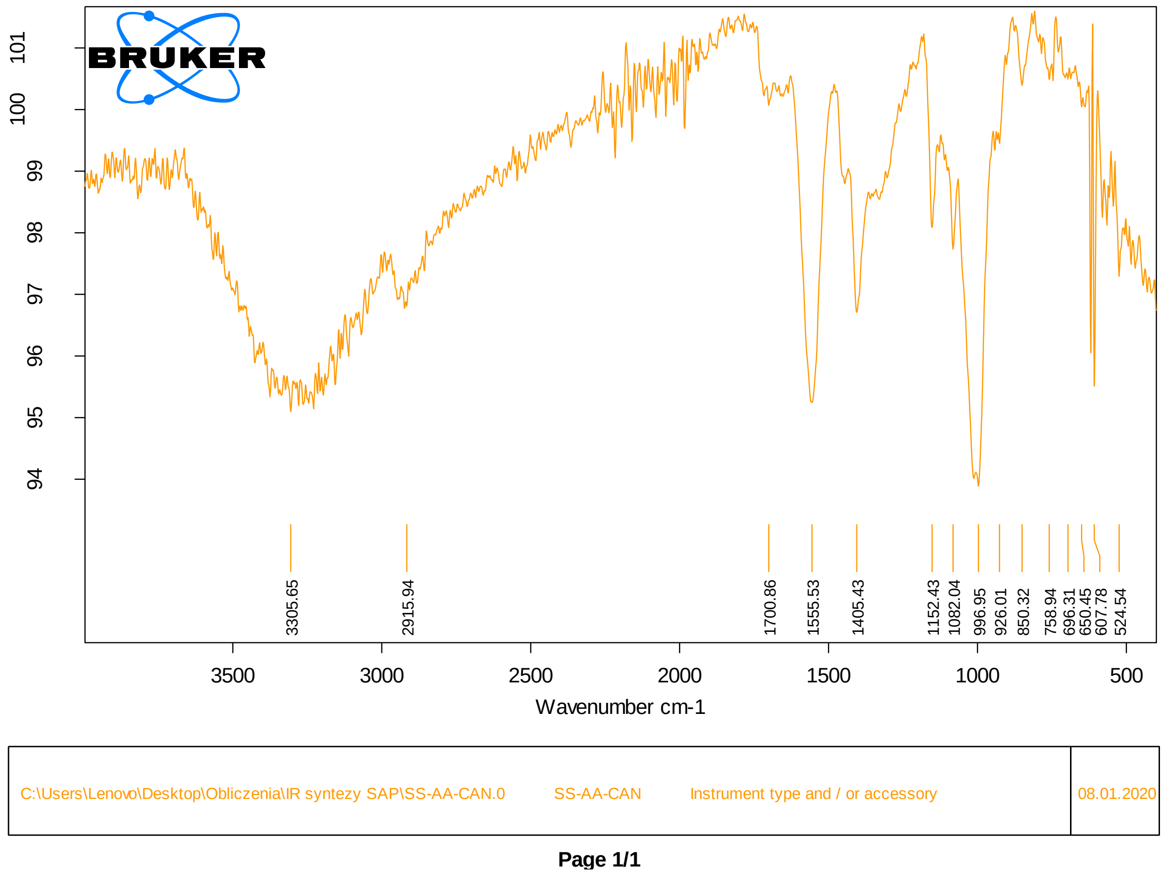

Figure S32. FTIR spectra of soluble starch-g-poly(acrylic acid) with ceric ammonium nitrate ((SS-g-PAA (CAN)).

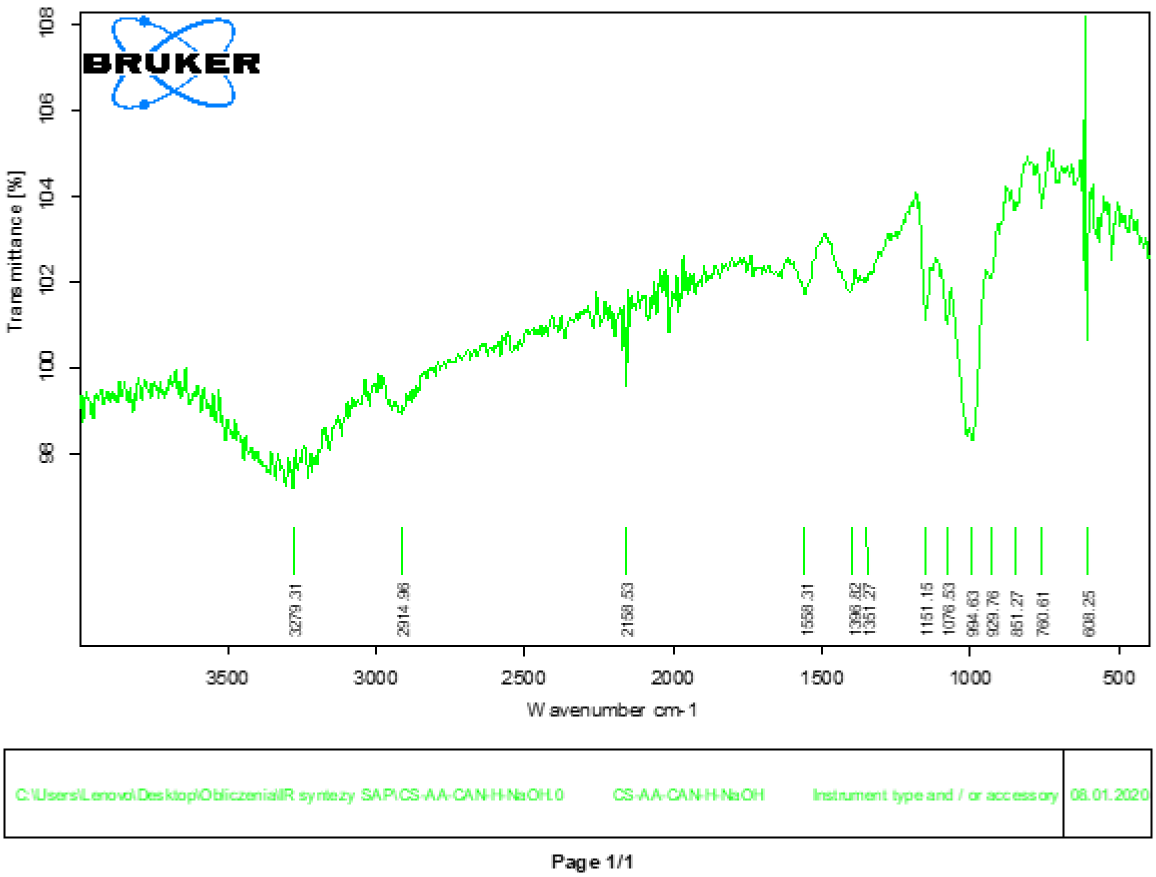

Figure S33. FTIR spectra of corn starch-g-poly(acrylic acid) with ceric ammonium nitrate, urea and NaOH (CS-g-PAA/U/NaOH/CAN).

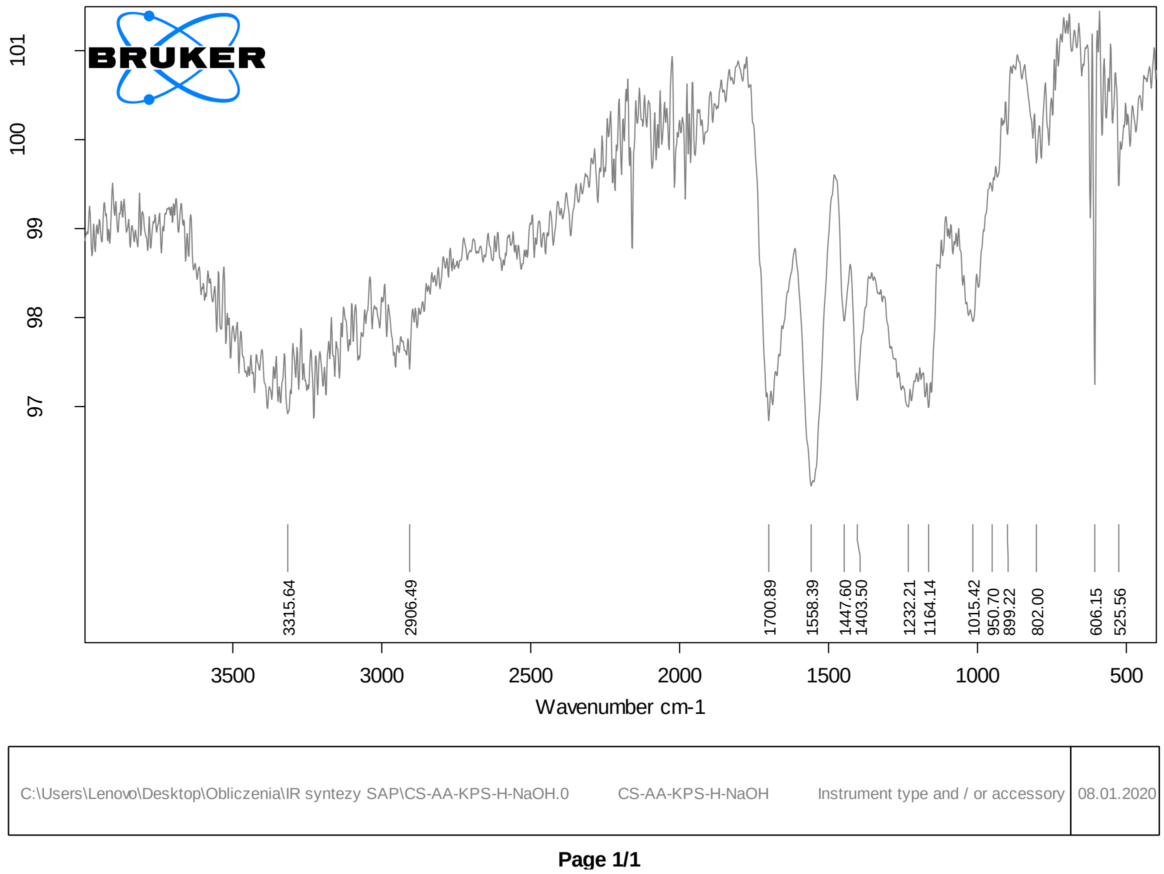

Figure S34. FTIR spectra of corn starch-g-poly(acrylic acid) with potassium persulfate, urea and NaOH (CS-g-PAA/U/NaOH/KPS).

3. Scanning electron microscope

Surface topography and size of superabsorbents used were tested using a scanning electron microscope manufactured by LEO Electron Microscopy Ltd. Cambridge, UK, model 1430 VP. Scanning electron microscopy was used to determine the shape, size, morphology, crosslinking density and porosity of superabsorbents.

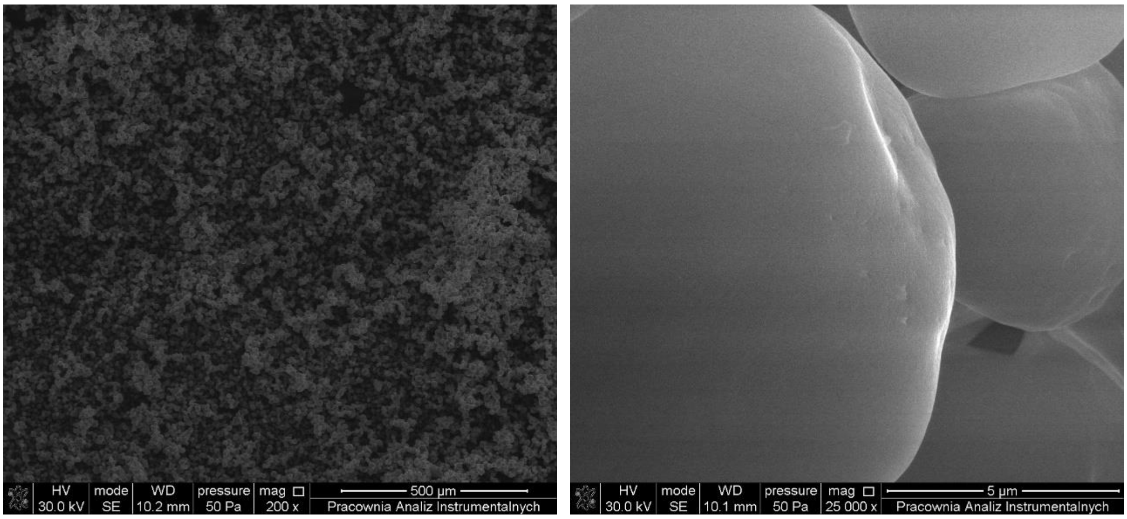

Figure S35. SEM images at 10000x and 25000x magnifications of corn starch (CS).

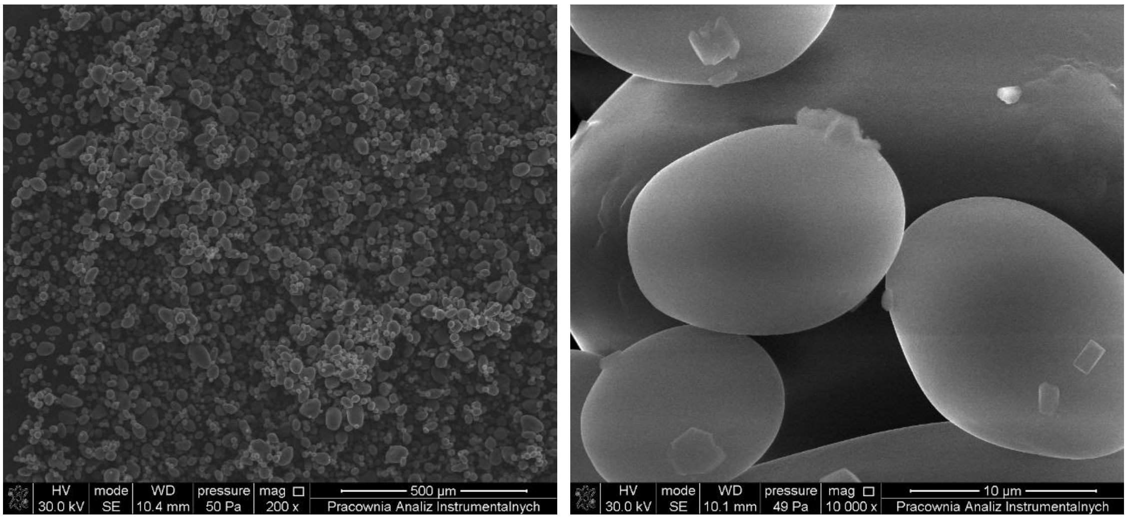

Figure S36. SEM images at 200x and 10000x magnifications of corn starch (CS).

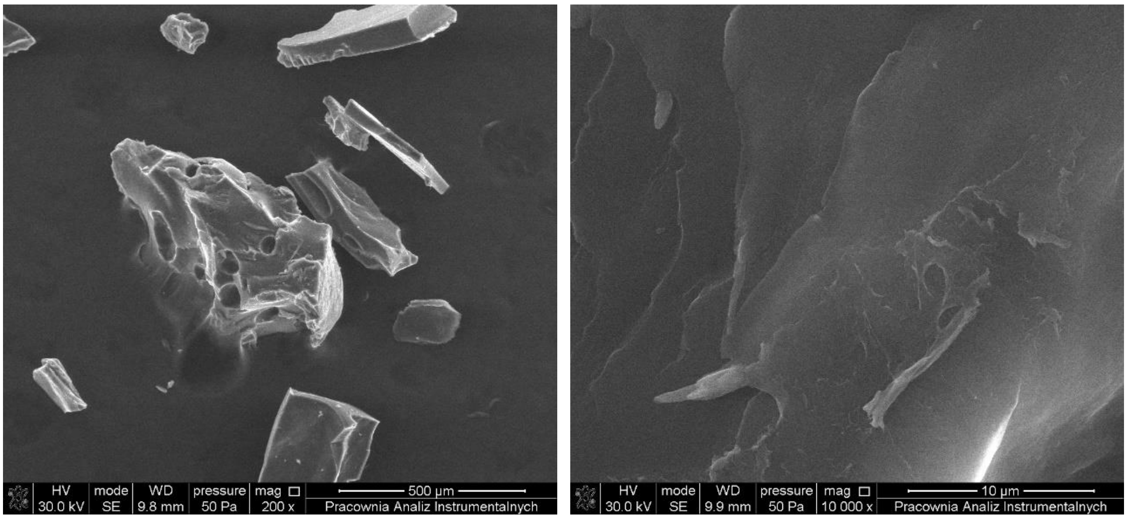

Figure S37. SEM images at 200x and 10000x magnifications of corn starch-g-poly(acrylic acid) with ceric ammonium nitrate, urea and NaOH (CS-g-PAA/U/NaOH/CAN).

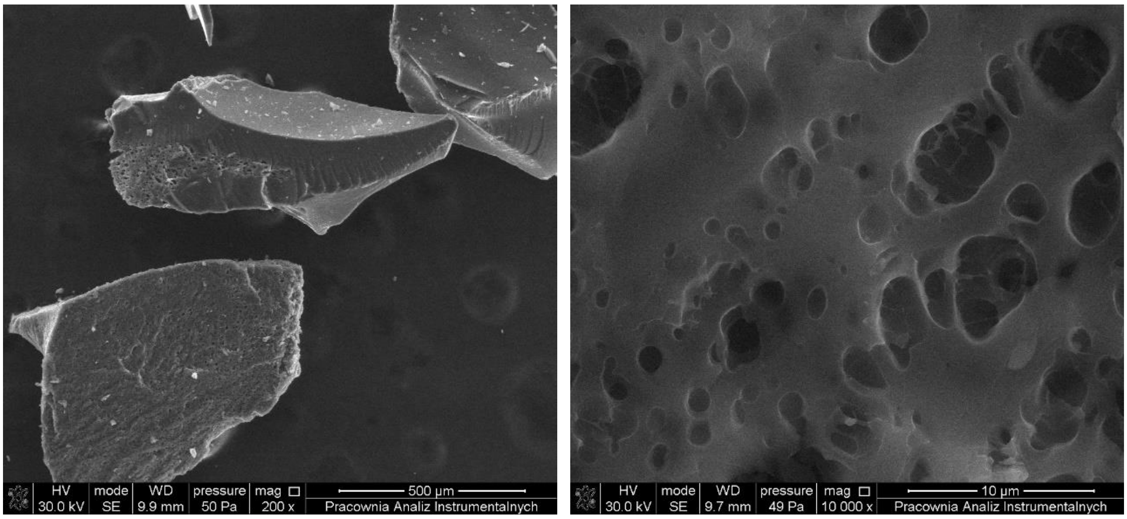

Figure S38. SEM images at 200x and 10000x magnifications of corn starch-g-poly(acrylic acid) with potassium persulfate, urea and NaOH (CS-g-PAA/U/NaOH/KPS).

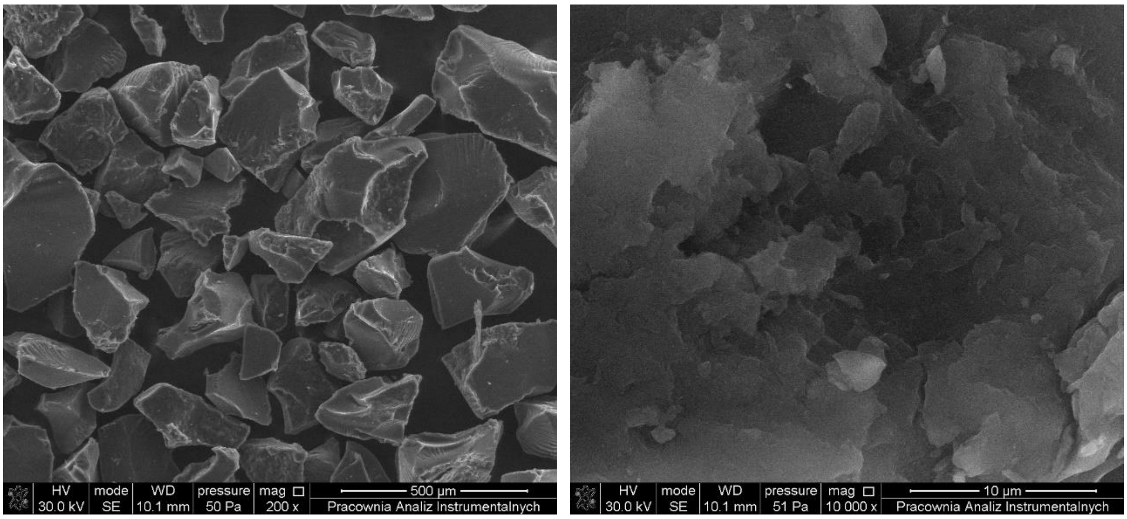

Figure S39. SEM images at 200x and 10000x magnifications of soluble starch-g-poly(acrylic acid) with ceric ammonium nitrate ((SS-g-PAA (CAN)).

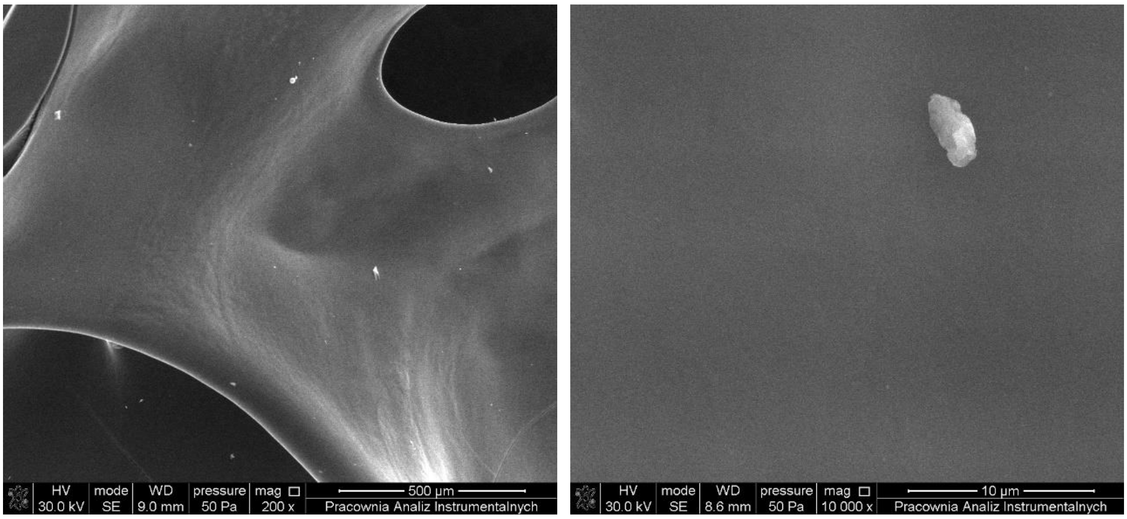

Figure S40. SEM images at 200x and 10000x magnifications of soluble starch-g-poly(acrylic acid) with ceric ammonium nitrate ((SS(12)-g-PAA (CAN)).

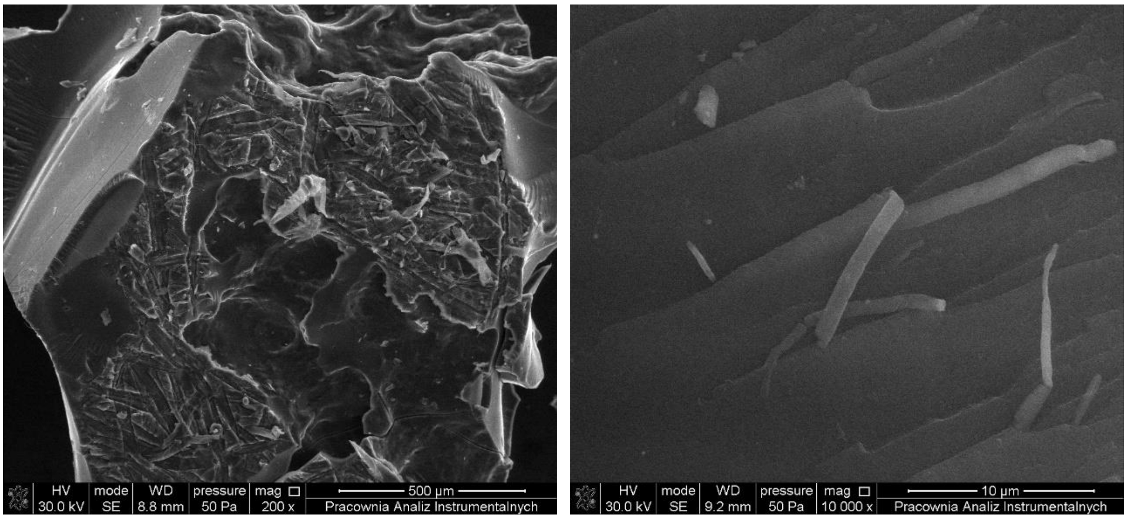

Figure S41. SEM images at 200x and 10000x magnifications of soluble starch-g-poly(acrylic acid) with ceric ammonium nitrate ((SS-g-PAA (12/CAN)).

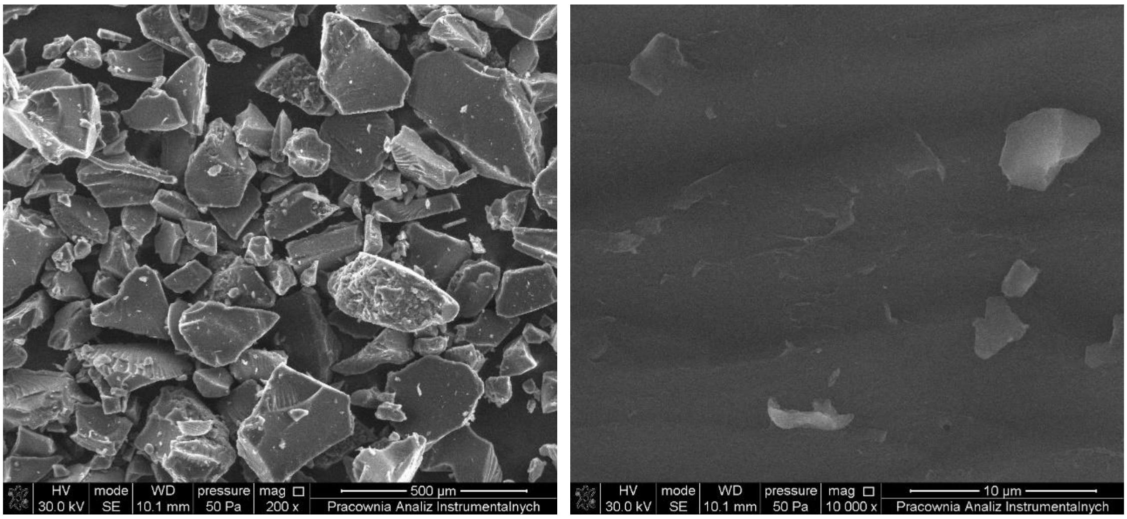

Figure S42. SEM images at 200x and 10000x magnifications of of soluble starch-g-poly(acrylic acid) with potassium persulfate (SS-g-PAA/KPS).
